# Tannin Reduces the Incidence of Polyspermic Penetration in Porcine Oocytes

**DOI:** 10.3390/antiox11102027

**Published:** 2022-10-14

**Authors:** Jing-Tao Sun, Jia-Hui Liu, Xi-Qing Jiang, Xin Luo, Jin-Dong Yuan, Qi Zhang, Xin-Yue Qi, Sanghoon Lee, Zhong-Hua Liu, Jun-Xue Jin

**Affiliations:** 1 Key Laboratory of Animal Cellular and Genetics Engineering of Heilongjiang Province, College of Life Science, Northeast Agricultural University, Harbin 150030, China; 2 Laboratory of Theriogenology, College of Veterinary Medicine, Chungnam National University, Daejeon 34134, Korea

**Keywords:** tannin, in vitro fertilization, polyspermy, organelle, pig

## Abstract

Tannin (TA) improves porcine oocyte cytoplasmic maturation and subsequent embryonic development after in vitro fertilization (IVF). However, the mechanism through which TA blocks polyspermy after IVF remains unclear. Hence, the biological function of organelles (cortical granule [CG], Golgi apparatus, endoplasmic reticulum [ER], and mitochondria) and the incidence of polyspermic penetration were examined. We found no significant difference in oocyte nuclear maturation among the 1 µg/mL, 10 µg/mL TA, and control groups. Moreover, 100 μg/mL TA significantly reduced 1st polar body formation rate compared to the other groups. Additionally, 1 and 10 μg/mL TA significantly increased the protein levels of GDF9, BMP15, and CDK1 compared to the control and 100 μg/mL TA groups. Interestingly, 1 and 10 μg/mL TA improved the normal distribution of CGs, Golgi, ER, and mitochondria by upregulating organelle-related gene expression and downregulating ER stress (*CHOP*) gene expression. Simultaneously, 1 and 10 μg/mL TA significantly increased the proportion of normal fertilized oocytes (2 pronuclei; 2 PN) and blastocyst formation rate compared to the control, as well as that of 100 μg/mL TA after IVF by upregulating polyspermy-related genes. In conclusion, TA during IVM enhances 2PN and blastocyst formation rates by regulating organelles’ functions and activities.

## 1. Introduction

The maturation process of mammalian oocytes is an extremely complicated biological event that includes both nuclear and cytoplasmic changes [1,2]. Nuclear maturation mainly involves chromosome segregation, which has been well-studied [3]. However, many controversial aspects of cytoplasmic maturation remain. Generally, cytoplasmic maturation involves the accumulation of mRNA, proteins, substrates, and nutrients required to achieve competence in oocyte development [4]. Additionally, the proper spatial and temporal dynamics of organelles, such as their localization, morphology, and biochemical properties during oocyte maturation, must occur for the oocyte to acquire high developmental potency [3]. Therefore, high-quality oocytes are essential for fertilization and preimplantation for embryo development. Nevertheless, the competence of embryo development was lower in the in vitro environment than in its in vivo counterparts after fertilization [5]. Polyspermy is one of the most commonly observed types of abnormal fertilization in mammalian oocytes. Polyploid formation resulting from polyspermic penetration induces abnormal early embryo development (early embryonic lethality or embryo developmental arrest) and even fails to develop to full-term with chromosomal abnormalities [6]. In humans, eggs are frequently fertilized by multiple sperm, which induces 10% of spontaneous abortions due to triploid [7]. A high incidence of polyspermy (50–90%) is also one of the major obstacles to the production of normal pig embryos in vitro [8,9,10,11,12]; polyspermy occurs more frequently in pigs than in other species. Thus, porcine oocytes are a natural and ideal model for studying polyspermic penetration after in vitro fertilization (IVF).

Tannins (TAs), powerful antioxidants, are a class of polyphenolic biomolecules found in a wide range of plants [13]. Moreover, they have astringent, antibacterial, and anti-enzymatic properties [14,15,16,17]. TA can effectively regulate ovarian follicle health and development by adjusting reproductive hormones, which participate in oocyte maturation and ovulation in the female reproductive system [18]. Additionally, TA modulates sperm capacitation and fertilization ability and improves freeze-thawed boar efficiency, thereby enhancing embryo development after IVF [19,20]. In a previous study, we demonstrated that TA supplementation during in vitro maturation (IVM) increased embryonic developmental competence by improving oocyte cytoplasmic maturation in pigs [13]. However, the mechanism through which TA blocks polyspermy during IVF remains unclear. Therefore, the objective of this study was to determine the effects of TA on intracellular organelle distribution and activity in oocytes, as well as the incidence of polyspermic penetration in pigs.

## 2. Materials and Methods

### 2.1. Chemicals

All chemicals and reagents were obtained from Sigma-Aldrich Chemical Company (St. Louis, MO, USA) unless otherwise stated.

### 2.2. Porcine Oocyte In Vitro Maturation

Porcine ovaries were transported from the local slaughterhouse (Harbin, Heilongjiang Province) to the laboratory at 30–34 °C in thermos, and cumulus–oocyte complexes (COCs) were obtained from small antral follicles (3–6 mm in diameter) using a syringe. Oocytes surrounded by a compact cumulus cell mass and homogeneous ooplasm were collected and washed with tissue culture medium-199 (TCM-199; Invitrogen, Carlsbad, CA, USA) containing 10 mM HEPES, 0.3% polyvinyl alcohol (PVA), and 1% penicillin–streptomycin, and placed into culture dishes containing IVM medium (TCM-199 containing 10% porcine follicular fluid, 0.91 mM sodium pyruvate, 10 IU/mL FSH and 10 IU/mL LH,) with 0, 1, 10, and 100 μg/mL TA (Sigma, 403040). The pooled 50 COCs/condition was then cultured in an incubator at 5% CO_2_, 100% relative humidity (RH), and 38.5 °C for 42 h [13]. After 42 h of IVM, cumulus cells were denuded from the oocytes by a pipette using 0.1% hyaluronidase. Subsequently, based on microscopic observations, the oocytes that extruded the first polar body were considered mature (metaphase II).

### 2.3. Detection of Embryo Development and Polyspermy after IVF

IVF was performed as described by Jin et al., with modifications [21]. After 42 h of IVM, 30 mature oocytes were placed into 50 μL of modified Tris-buffer medium (mTBM), and fresh semen was prepared for IVF. Boar semen was washed three times in Dulbecco’s phosphate-buffered saline (PBS) with 0.1% bovine serum albumin (BSA) and centrifuged at 2000 rpm for 4 min. The semen pellet was suspended in 1 mL of mTBM as a final concentration of 1.0 × 10^6^ spermatozoa/mL and placed into 50 μL mTBM droplet with matured oocytes. Immediately before insemination, sperm motility was assessed and >80% motile sperm was used in each experiment. Then, the samples were co-incubated for 6 h under 5% CO_2_ atmosphere at 38.5 °C. Subsequently, the presumptive zygotes were washed three times and transferred into an in vitro culture medium, porcine zygote medium 3 (PZM3), then incubated at 38.5 °C at 100% RH and 5% CO_2_ for 7 days. The zygotic pronucleus was detected by nuclear staining without the zona pellucida in 10 μg/mL Hoechst-33342 for 4 min after an additional 12 h of IVF. Moreover, cleavage and blastocyst rates were evaluated on day 2 and day 7, respectively. Blastocysts were collected on day 7 and stained with 10 μg/mL Hoechst-33342 for 4 min in order to count the total cell number per blastocyst.

### 2.4. Immunofluorescence (IF) Staining

Immunofluorescence staining was performed as previously described by Lee et al. [22]. Porcine oocytes were washed three times in PBS containing 0.1% PVA and fixed with 4% paraformaldehyde (PFA). After washing three times in PBS, the oocytes were placed in 1% Triton X-100 for 30 min and incubated in 2% BSA-PBS to block non-specific sites overnight at 4 °C. Porcine oocytes were incubated with rabbit polyclonal antibodies against GDF9 (ab93892; Abcam, Cambridge, UK) and BMP15 (PA5-34401; Invitrogen) at 37 °C for 2 h. The samples were washed three times in 2% BSA and then incubated with a goat anti-rabbit fluorescein isothiocyanate-conjugated secondary antibody (1:200; Jackson Immuno Research Laboratories Inc., West Grove, PA, USA) for 2 h. Oocytes were mounted on glass slides and the images were captured using an epifluorescence microscope (TE2000-S; Nikon). Fluorescence intensities were measured using ImageJ software (version 1.46 r; National Institutes of Health).

### 2.5. Staining of CG, Golgi, ER, and Mitochondria in Oocytes

After 42 h of IVM, mature porcine oocytes were fixed with 4% PFA for 30 min, washed, blocked with 0.3% BSA and 1 M glycine solution, and permeabilized in 0.1% Triton X-100 for 5 min. After washing three times, the oocytes were incubated with 100 µg/mL fluorescein isothiocyanate conjugated to peanut agglutinin (FITC-PNA, L7381; Sigma) for 30 min. The labeled oocytes were mounted, and the cortical granule (CG) distribution was observed under a laser-scanning confocal microscope (TE2000-U; Nikon). The distribution of CGs was classified as below (Figure 2A), according to a method described previously [23]. The active CGs distribution was assessed through several optical sections, each with a thickness of 3 μm [24]: (a) peripheral distribution: CGs were adjacent to the plasma membrane; (b and c) homogeneous distribution: CGs were distributed to half of the plasma membrane; and (d) cortical distribution: CGs were distributed throughout the cytoplasm and the plasma membrane.

For Golgi apparatus and endoplasmic reticulum (ER) staining, matured oocytes were fixed in 4% PFA for 10 min, washed three times with pre-cooled 0.2% PVA-PBS, and then placed in 5 mM NBD C6-Ceramide (C049-E; GeneCopoeia, Heidelberg, Germany) and 1 μM ER-Tracker Red (E34250; Invitrogen) for 30 min at 4 °C in the dark. The samples were blocked in 2% BSA-PBS for 1 h at 23 °C. The labeled oocytes were mounted and the images were captured using an epifluorescence microscope (TE2000-S; Nikon). Fluorescence intensities were measured using ImageJ software (version 1.46 r; National Institutes of Health). The distributions of Golgi and ER were classified as below (Figures 2B and 3A) according to methods described previously [23,25]: (a) normal distribution, where the organelles were homogeneously distributed in the cytoplasm; (b–d) abnormal distribution, where half of cytoplasmic distribution, large granulations of mitochondria distributed throughout the cytoplasm, large cavity shapes (Figure 2B(d)), or plasma membrane distributions (Figure 3A(d)) were observed.

The mitochondria of the oocytes were stained using MitoTracker Green FM (M7514; Invitrogen). Mature oocytes were blocked with 2% BSA-PBS for 2 h at 37 °C, washed three times in 0.2% PVA-PBS, and stained with 200 nM MitoTracker Green for 30 min at room temperature. After washing three times, the samples were mounted and the images were captured using an epifluorescence microscope (TE2000-S; Nikon). Fluorescence intensities were measured using ImageJ software (version 1.46 r; National Institutes of Health). The mitochondrial distribution was modified according to the method described by Pawlak et al. [26]. (a) Homogeneous fine, with small granulations spread throughout the cytoplasm; (b–d) heterogeneous distribution, with large granulations spread throughout the cytoplasm, or peripherical and central granular aggregations of mitochondria (Figure 3B).

### 2.6. Real-Time Polymerase Chain Reaction

Total mRNA was extracted from 100 oocytes in each group using a TRIzol reagent (Invitrogen), according to the manufacturer’s protocol. The complementary DNA (cDNA) was produced using amfiRivert cDNA Synthesis Platinum Master Mix (GenDEPOT, Barker, TX, USA). A PCR reaction mix (Micro-Amp Optical 96-Well Reaction Plate, Singapore) was prepared by adding 1 µL of cDNA, 0.4 µL (10 pmol/µL) of forward and reverse primers, 8.2 µL of nuclease-free water (NFW; Ambion, Austin, TX, USA), and 10 µL of SYBR Premix Ex Taq (TaKaRa, Otsu, Japan), and then amplified on a StepOneTM Real-Time PCR System (Applied Biosystems, Waltham, MA, USA). The amplification protocol included an initial denaturation step for 10 min at 95 °C, followed by 40 cycles consisting of denaturation for 15 s at 95 °C, annealing for 1 min at 60 °C, and extension for 1 min at 72 °C. The expression of each target gene was quantified relative to the reference genes *GAPDH* and *RN18S,* using the equation R = 2 ^−∆∆Ct^. Primer sequences are shown in Table 1.

### 2.7. Western Blotting (WB)

After 42 h of IVM, mature oocytes from each group were washed three times in PBS and lysed in 60 μL of lysis buffer (150 mM NaCl, 1 mM EDTA, 20 mM glycerol phosphate and HEPES, 10% glycerol, 1% Triton X-100, and 2 mM EGTA) containing 1% phenylmethylsulfonyl fluoride (100 nM; Beyotime, Haimen, China) for 3 h. The samples were then boiled for 5 min at 100 °C. Approximately 1 μg of total protein was loaded onto a 12% ExpressPlusTM PAGE Gel (GenScript, Nanjing, China). The gel was then transferred onto a nitrocellulose (NC) membrane (Millipore, Bedford, MA, USA). Non-specific sites were blocked using Tris-buffered saline plus 0.05% Tween 20 (TBST) overnight in primary antibodies (CDK1, ab18, Abcam; p-CKD1 p34, pY15.44, Santa Cruz) at 4 °C. The NC membrane was washed three times with TBST, incubated with horseradish peroxidase-conjugated secondary antibodies, incubated in SuperSignal West Femto Maximum Sensitivity substrate (Thermo Scientific, Waltham, MA, USA) for 10 min, and then scanned using a Fujifilm LAS-3000 Imaging System (Fuji, Edison, NJ, USA). The bands were quantified using the ImageJ version 1.46 r software.

### 2.8. Statistical Analyses

Data are presented as mean ± standard mean of error (SEM). Significant differences were determined using Duncan’s test, following parametric one-way analysis of variance (ANOVA) with the statistical software SPSS (version 19.0; SPSS Inc., Chicago, IL, USA). Differences with *p* < 0.05 were considered statistically significant.

## 3. Results

### 3.1. Effects of TA on Porcine Maturation

In order to evaluate porcine oocyte nuclear maturation, we supplemented the oocytes with different concentrations of TA during IVM. The results showed that there was no significant difference in nuclear maturation among the groups (control: 81.1 ± 3.8%, 1 μg/mL TA: 86.6 ± 2.8%, and 10 μg/mL TA: 84.6 ± 2.8%; Table 2). However, 100 μg/mL TA (60.5 ± 3.4%) significantly reduced nuclear maturation compared to the other groups (*p* < 0.05).

**Figure 1:**
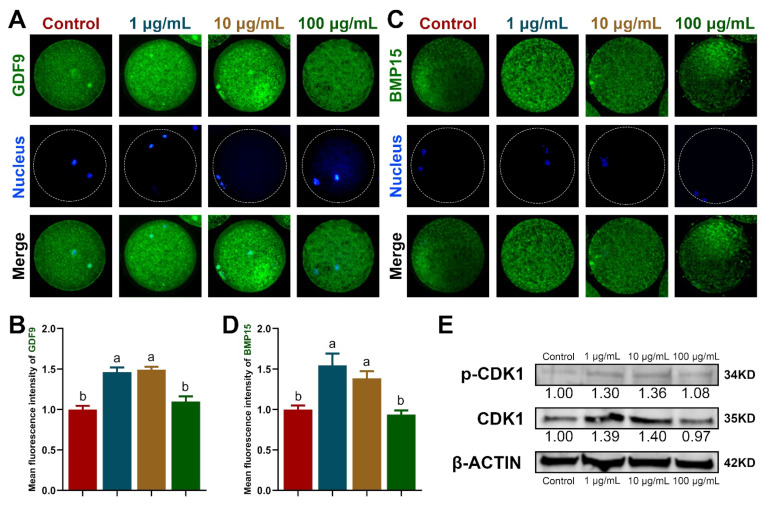
Detection of GDF9 and BMP15 proteins by immunofluorescence staining and detection of p-CKD1 and CKD1 proteins by Western blot in oocytes. (**A**,**B**) GDF9 protein expression. (**C**,**D**) BMP15 protein expression. (**E**) p-CDK1 and CDK1 protein expression. Within the same indicator, bars with different letters indicate a significant difference (*p* < 0.05). Results are shown as the average ± SEM of at least three repeats of independent experiments. A total of 30 oocytes were analyzed per group in each replicate.

Additionally, we measured the protein levels of GDF9 and BMP15 in the oocytes. As shown in Figure 1A–D, 1 and 10 μg/mL TA significantly increased the protein levels of GDF9 and BMP15 compared with the control and 100 μg/mL TA (*p* < 0.05). The WB results showed that p-CDK1 levels were significantly upregulated up to 1.30-fold and 1.36-fold in response to 1 and 10 μg/mL TA, respectively (*p* < 0.05; Figure 1E). However, there was no significant difference in the p-CDK-1 levels between the 100 μg/mL TA control groups. In line with these results, the level of CDK1 was significantly increased by 1.39-fold and 1.40-fold in the 1 and 10 μg/mL TA groups, respectively, compared to that in the control group (*p* < 0.05).

### 3.2. CGs and Golgi Distribution Rate and Activity

**Figure 2:**
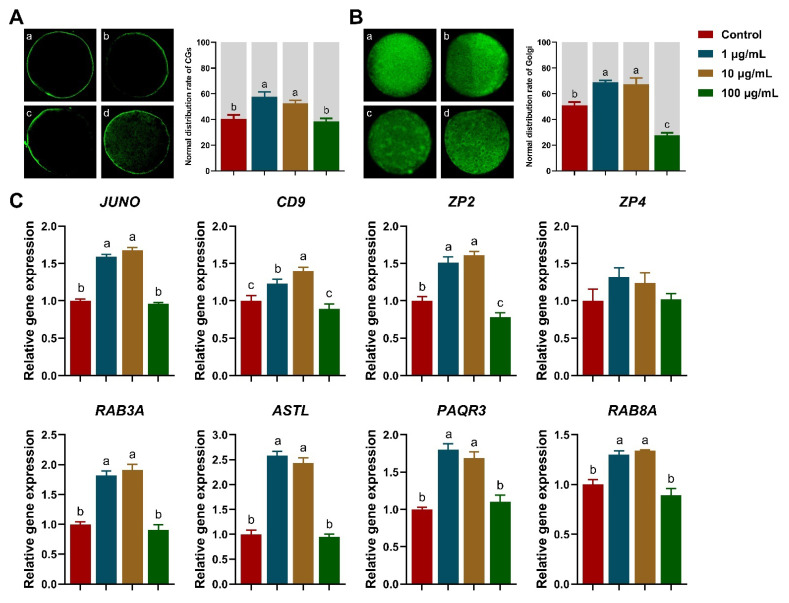
Effects of TA on the distribution of the cortical granule (CG) and Golgi apparatus (Golgi) in porcine oocytes. (**A**) CG distribution: (**a**) Peripheral CG distribution (normal); (**b**,**c**) homogeneous distribution (abnormal); (**d**) cortical distribution (abnormal). (**B**) Golgi distribution: (**a**) homogeneous distribution (normal); (**b**–**d**) unequal distribution (abnormal). (**C**) Polyspermy, CG, and Golgi-related gene expression. Within the same indicator, bars with different letters are significantly different (*p* < 0.05). Results are shown as the average ± SEM of at least three repeats of independent experiments. A total of 30 oocytes were analyzed per group in each replicate.

During oocyte maturation, 1 and 10 μg/mL TA supplementation significantly increased the normal distribution rate of CGs compared to that of the control and 100 μg/mL TA groups (*p* < 0.05; Figure 2A). Moreover, the CG-related gene (*RAB3A* and *ASTL*) expression was significantly upregulated in the 1 and 10 μg/mL TA groups (*p* < 0.05; Figure 2C).

In line with CGs results, the normal distribution rate of Golgi was upregulated to 69.11 ± 1.38% (1 μg/mL TA) and 67.55 ± 4.86% (10 μg/mL TA) compared with that of the control (51.11 ± 2.54%; *p* < 0.05), and 100 μg/mL TA (27.84 ± 1.83%) showed a reduction in the normal distribution rate compared to the control *(p* < 0.05; Figure 2B). Moreover, Golgi activity was significantly enhanced by 1 and 10 μg/mL TA supplementation, as shown by the increased mRNA expression of *PAQR3* and *RAB8A (p* < 0.05; Figure 2C).

### 3.3. Distribution Rates of ER and Mitochondria

**Figure 3:**
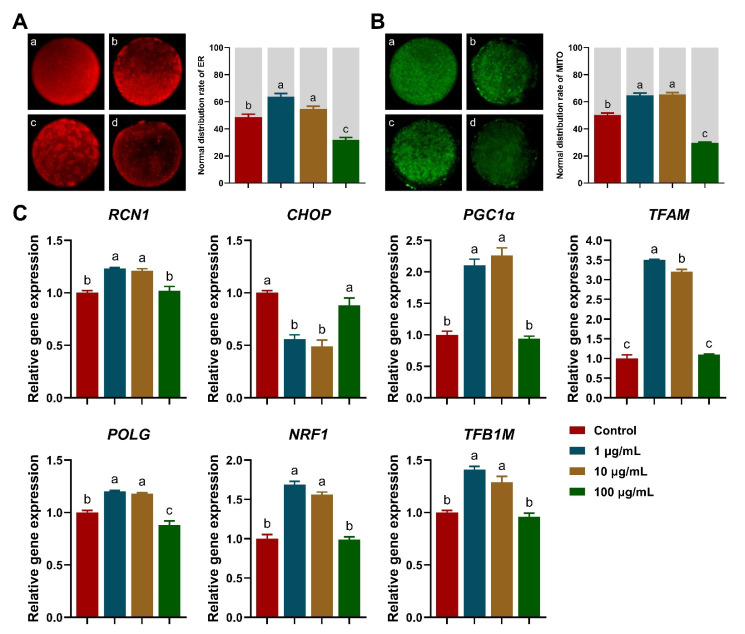
Effects of TA on the distribution of endoplasmic reticulum (ER) and mitochondria in porcine oocytes. (**A**) ER distribution: (**a**) homogeneous distribution (normal); (**b**–**d**) unequal distribution (abnormal). (**B**) Mitochondria distribution: (**a**) peripheral (normal); (**b**–**d**) cortical or diffused (abnormal). (**C**) ER and mitochondrial biogenesis-related gene expression. Within the same indicator, bars with different letters are significantly different (*p* < 0.05). Results are shown as the average ± SEM of at least three repeats of independent experiments. A total of 30 oocytes were analyzed per group in each replicate.

Abnormal distribution rates of ER and mitochondria were significantly decreased upon 1 and 10 μg/mL TA supplementation compared to the control (*p* < 0.05; Figure 3A,B), and 100 μg/mL TA exhibited the lowest proportion of ER and mitochondria, with a normal distribution (*p* < 0.05). Moreover, ER activity and stress were significantly upregulated with increased *RCN1* mRNA expression, and downregulated with decreased *CHOP* mRNA expression upon 1 and 10 μg/mL TA supplementation, respectively (*p* < 0.05; Figure 3C). Mitochondrial biogenesis was drastically improved with 1 and 10 μg/mL TA supplementation with increased *PGC1α*, *TFAM*, *POLG*, *NRF1,* and *TFB1M* mRNA expression (*p* < 0.05; Figure 3C).

### 3.4. Polyspermic Penetration Incidence and Embryo Development Competence after IVF

As shown in Table 3, the 2PN (2 pronuclei; normal fertilization) formation rate was significantly increased in 1 and 10 μg/mL TA groups compared to that in the control and 100 μg/mL TA groups (*p* < 0.05). Additionally, anti-polyspermic genes, such as *JUNO*, *CD9,* and *ZP2,* were significantly upregulated with 1 and 10 μg/mL TA supplementation (*p* < 0.05; Figure 2C). Coincidentally, the blastocyst formation rate was significantly improved with 1 and 10 μg/mL TA supplementation (*p* < 0.05; Table 4), but there was no significant difference in the cleavage rate.

## 4. Discussion

In pigs, the incidence of polyspermic penetration has been increasingly recognized to depend on sperm quality during IVF [11]. However, researchers have recently focused on the quality of oocytes [13,27,28] because the pattern and dynamics of the spatial and temporal distribution of organelles during oocyte maturation are crucial for oocyte quality [3], and, consequently, normal fertilization [11]. TA supplementation increases the normal fertilization rate (2PN formation) by modulating capacitation and sperm fertilization ability [19]. In addition, our previous study demonstrated that TA supplementation during IVM increased embryo developmental competence after PA, IVF, and SCNT by improving oocyte cytoplasmic maturation in pigs [13]. In this study, we demonstrated that TA could positively regulate organelle activity and distribution during IVM, and thereby decrease the incidence of polyspermic penetration in porcine IVF, resulting in an overall increase in the efficiency of standard IVF protocols.

Maturation is one of the terminal processes in the development of oogenesis. Nuclear and cytoplasmic maturation, which take place during the last stages of oocyte maturation, are critical determinants of oocyte quality and subsequent embryonic development [29,30]. Briefly, oocyte nuclear maturation is chromosomal segregation, which includes the acquisition of meiotic competence, meiotic resumption, and completion of meiosis I, as well as the maintenance of metaphase II arrest, as evidenced by the extrusion of the 1st polar body [31]. Nuclear maturation is associated with the maturation-promoting factor (MPF), which plays a pivotal role in modulating the meiotic cell cycle by regulating the complex of CDK1 and CYCLIN B [32]. Although the first polar body extrusion was not significantly different between the 1 and 10 μg/mL TA groups, the protein levels of p-CDK1 and CDK1 were increased by 1.3-fold compared to the control. In our previous study, we found that TA upregulated *CYCLIN B1* and *CDK1* mRNA and protein expression in porcine oocytes [13]. Additionally, high MPF activity is present in high-quality, mature porcine oocytes [33]. Therefore, we speculate that TA supplementation might improve oocyte quality by improving cytoplasmic maturation, as shown by the expression of two genes with maternal effects (GDF9 and BMP15), although nuclear maturation was not affected.

Cytoplasmic maturation is one of the key factors that determine the quality of oocytes, including the accumulation of mRNA, proteins, substrates, and nutrients, as well as the arrangement and maturation of organelles, especially CGs, Golgi, ER, and mitochondria [34]. However, oxidative stress is mainly caused by production of intracellular reactive oxygen species (ROS), which impair proteins and organelles to maintain cell health. CGs are secretory organelles derived from the Golgi apparatus and stored in the cortex of unfertilized oocytes [35]. Following fertilization, CGs undergo exocytosis in order to release their contents into the perivitelline space [36]. Austin showed that these granules almost completely disappeared after spermatozoon penetration [37]. Therefore, the fusion of cortical granules with the oocyte plasma membrane during oocyte maturation is the most effective event to block sperm entry [34]. Rab3A plays an active role in CGs exocytosis in murine eggs during cortical reactions [35]. It has been reported that inhibition of endogenous Rab3A function by microinjecting a polyclonal antibody abolished CGs exocytosis in mouse oocytes [35]. ZP2 is proteolytically cleaved after gamete fusion to prevent polyspermy, which is associated with exocytosis of CGs in oocytes [38]. Furthermore, Ren et al. found that Astl, a CGs marker protein, is critical for the distribution and migration of CGs in oocytes [39]. Therefore, Astl deletion in mouse oocytes resulted in failure to cleave ZP2 and continuation of ZP binding and penetration [38]. Moreover, Astl is downregulated in the fertilization of post-ovulatory aged mouse oocytes, thereby resulting in lower embryo development competence [40]. Taken together, the function and distribution of Golgi and CGs might be critical for cytoplasmic maturation and quality of oocytes, which protect from polyspermic incidence, thereby improving subsequent embryonic development.

Another way to prevent polyspermy is the fusion of the first sperm, which causes depolarization of the membrane and shedding of the egg’s sperm receptor Juno [41]. Female mice lacking *Juno* are infertile, and *Juno*-deficient eggs do not fuse with normal sperm. Moreover, Cd9, a partner of Juno, concomitantly accumulates in the adhesion area, but without Cd9, the recruitment kinetics of Izumo1 (testis immunoglobulin superfamily type 1 protein) is accelerated [42]. In this study, we found that polyspermy-related genes (*JUNO*, *CD9,* and *ZP2*) were upregulated by 1 and 10 μg/mL TA supplementation. Furthermore, CGs and Golgi-related genes (CGs: *RAB3A* and *ASTL*; Golgi: *PAQR3* and *RAB8A,* respectively) were drastically increased with 1 and 10 μg/mL TA supplementation, resulting in higher rates of normal distribution of CGs and Golgi. Interestingly, the proportion of normal fertilized oocytes (2PN) was improved with 1 and 10 μg/mL TA supplementation by reducing the polyploid (>3PN) rate. Additionally, our results showed that although the cleavage and total cell numbers/blastocyst were not affected, the blastocyst formation rate was significantly increased by improving oocyte quality during IVM media supplementation with 1 and 10 μg/mL TA. Therefore, our results demonstrated that TA supplementation effectively prevents polyspermic fertilization by production, migration, and distribution of CGs, which is critical to cortical reaction and zona hardening, and thereby effectively block the penetration of the second sperm into the oocyte and improve the competence of the embryo development.

Following experiments, we examined the other organelles’ function and distribution, as well as their related gene expressions, including the ER and mitochondria. Our results showed that TA could improve the normal distribution rate of ER, increase the *RCN1* expression (inhibiting ER stress-induced apoptosis), and decrease the *CHOP* expression (inducing ER stress and apoptosis). ER plays several roles in the cell, including protein synthesis and lipid metabolism [43]. ER stress triggers ROS signaling, changes the redox status, and regulates the antioxidant defect [44]. Therefore, relief of ER stress decreases apoptosis and improves oocyte maturation and embryo development competence [45]. The ER also acts as the major storage area for calcium ions (Ca^2+^), thus regulating intracellular Ca^2+^ homeostasis [46]. ER stress induces calcium-dependent permeability transition and mitochondrial outer membrane permeabilization [47]. Moreover, the cortical reaction is a calcium-dependent exocytotic process, which participates in the reduction in incidences of polyspermic penetration [48]. Therefore, functional homeostasis of the ER is indispensable factor for CGs exocytosis, forbidding the penetration of the second sperm into the oocyte.

Mitochondrial biogenesis is modulated at the transcriptional, translational, and post-translational levels [17], and PGC1α is a key regulator of mitochondrial biogenesis [18]. Mitochondrial biogenesis is crucial for the development of oocytes and the process of selective inheritance, which regulates the transmission of harmful mtDNA mutations [49]. Moreover, NRF and downstream TFAM are the main regulators of mitochondrial biosynthesis. In a previous study, we found that the improvement of PGC1α expression resulted in Nrf2 increasing, which synthesized antioxidant enzymes GSH and SOD1, scavenged the ROS, and promoted mitochondrial bio-function and biogenesis, thereby improving oocyte quality by TA supplementation during porcine IVM [13]. In this study, the normal mitochondrial distribution rate and its biogenesis-related gene expression (*PGC1α*, *TFAM*, *POLG*, *NRF1,* and *TFB1M*) increased with 1 and 10 μg/mL TA supplementation in porcine oocytes. In contrast, severe oxidative stress triggered a decrease in cellular mitochondrial content through the suppression of mitochondrial biogenesis, as well as mitochondrial function [46]. In conclusion, there are indispensable functions and activities between different organelles during oocyte maturation and embryo development. Therefore, these results indicate that there is a small likelihood of polyspermy by improving oocyte quality following TA treatment, due to the enhanced function of the cytoplasmic organelles.

## 5. Conclusions

In the current study, we demonstrated that TA improved the blastocyst formation rate by effectively improving oocyte quality and eliminating the incidence of polyspermic penetration, which is mainly modulated by TA-induced ooplasmic improvement, especially the normal distribution rate of cytoplasmic organelles and their functions and activities. These results provide the basis for the rational development of new fertility treatments.

## Figures and Tables

**Table 1:** Primer sequences used for real-time PCR.

Genes	Primer Sequences (5′–3′)	Product Size	Accession No.
*GAPDH*	F: GTCGGTTGTGGATCTGACCT R: TTGACGAAGTGGTCGTTGAG	207	NM_001206359
*RN18s*	F: TCCAATGGATCCTCGCGGAA R: GGCTACCACATCCAAGGAAG	149	NR_046261
*JUNO*	F: CTGACTGCCGCACATCCTAC R: GATCTTCTCGCACAGGTCGG	146	XM_013979283
*CD9*	F: TTGCCATTGAAATAGCCGCG R: AGGCTTGAGAGTACGTCCCT	229	NM_214006
*ZP2*	F: GAGTGACTCGCTACTCGCAA R: GTCACAGGATCTGCCACACA	124	NM_213848
*ZP4*	F: GACCCACCCTGGACCTCTTA R: GCTCCCGAAGCAATTTCACC	107	NM_214045
*RAB3A*	F: GCCTTTGTCAGCACTGTAGGA R: ATAGGCTGTCGTGATGGTCC	126	NM_001123179
*ASTL*	F: GTGCTCTCCAGCAAGTACGA R: CCCAGACATGGGTACGATGG	138	XM_003481133
*PAQR3*	F: TATGTGATCGCTGTCCTCGC R: GACCGCAAGGATGTGCCATA	117	XM_003129372
*RAB8A*	F: TGGCGAAGACCTACGATTACC R: GAGAAGCGGAACAGGACACA	85	NM_001243802
*RCN1*	F: CCTCCCGCAAGACTATGACC R: AGCTTGGCTCCCAACAAACA	133	XM_003353917
*CHOP*	F: AGGCCTGGTATGAGGACCTG R: GCTGTGCCACTTTCCTTTCA	339	NM_001144845
*PGC1α*	F: TTCCGTATCACCACCCAAAT R: ATCTACTGCCTGGGGACCTT	137	NM_213963
*TFAM*	F: TCCGTTCAGTTTTGCGTATG R: TTGTACACCTGCCAGTCTGC	240	NM_001130211
*POLG*	F: ATGGGCCTTACAACGAGGTG R: GGCCTTCCTCATCAAAGGCT	300	XM_001927064
*NRF1*	F: TTCTGCTGTGGCTGATGGAG R: AGTGCCATGGTGACTGTAGC	174	XM_021078993
*TFB1M*	F: TGGCAGAACCCAGATGACAC R: GGGCCATGATGGAAAGACGA	101	NM_001128475

F, forward primer; R, reverse primer.

**Table 2:** Effects of tannin on porcine oocyte maturation during in vitro maturation.

Tannin Concentration, μg/mL	No. of COCs Cultured	No. of Oocytes with (Mean ± SEM, %)					
		Degenerated (%)		Immature (%)		Metaphase II (%)	
0	483	36	(7.3 ± 2.3)	56	(11.6 ± 2.5) ^b^	391	(81.1 ± 3.8) ^a^
1	488	32	(6.6 ± 1.6)	33	(6.8 ± 1.8) ^b^	423	(86.6 ± 2.8) ^a^
10	472	33	(7.0 ± 1.7)	38	(8.4 ± 1.5) ^b^	401	(84.6 ± 2.8) ^a^
100	494	48	(9.8 ± 2.0)	147	(29.7 ± 2.9) ^a^	299	(60.5 ± 3.4) ^b^

^a, b^ Values with different superscript letters indicate statistical significance (*p* < 0.05). COCs, Cumulus–oocyte complexes. In vitro oocyte maturation was performed in 10 replicates.

**Table 3:** Effects of tannin on polyspermic penetration incidence in porcine oocytes.

Tannin Concentration, μg/mL	No. of MII Oocytes	No. of Fertilization Status (Mean ± SEM, %)					
		Normal Fertilized (%)		≥3PN (%)		Nonfertilized (%)	
0	445	142	(30.3 ± 7.4) ^b^	232	(53.9 ± 9.4) ^a^	71	(15.7 ± 4.6) ^b^
1	443	281	(63.0 ± 5.9) ^a^	129	(30.1 ± 8.6) ^b^	33	(6.9 ± 3.9) ^b^
10	441	255	(58.4 ± 6.5) ^a^	153	(34.2 ± 3.8) ^ab^	33	(7.4 ± 3.0) ^b^
100	438	92	(20.5 ± 4.9) ^b^	124	(28.0 ± 2.6) ^b^	222	(51.5 ± 7.1) ^a^

^a, b^ Values with different superscript letters indicate statistical significance (*p* < 0.05). PN, pronuclei. The polyspermic penetration incidence was performed in 3 replicates.

**Table 4:** Effects of tannin on embryo development competence after in vitro fertilization.

Tannin Concentration, μg/mL	No. of Embryos Cultured	No. of Embryos Developed to (Mean ± SEM, %)				Total Cell Numbers /Blastocyst
		**Cleavage (%)**		**Blastocyst (%)**		
0	314	259	(82.1 ± 4.1)	50	(16.0 ± 0.5) ^b^	65.7 ± 4.1 ^a^
1	312	256	(81.9 ± 3.4)	70	(22.4 ± 1.5) ^a^	63.9 ± 7.7 ^a^
10	324	277	(85.1 ± 3.7)	83	(25.6 ± 1.1) ^a^	63.2 ± 5.9 ^a^
100	265	198	(74.8 ± 4.0)	36	(13.8 ± 1.4) ^b^	50.7 ± 4.2 ^b^

^a, b^ Values with different superscript letters indicate statistical significance (*p* < 0.05). In vitro embryo culture was performed in 7 replicates.

## Data Availability

The data are contained within the article.
